# Supplementary material for: Countercurrent heat exchange and thermoregulation during blood-feeding in kissing bugs
Source: eLife. 2017 Nov 21;6:e26107. doi: 10.7554/eLife.26107 (PMC5697934; doi:10.7554/eLife.26107)
Supplement: Supplementary file 1. — (See also Figure 2—figure supplement 1). From top to bottom, Ta = 16°C, 21°C, 26°C or 31°C. [file elife-26107-supp1.docx]

**Supplementary file 1**

*Two-way* ANOVA tables for the analysis of the impact of the temperature of the blood (*T_blood_*) and the environmental temperature on the different body parts of *Rhodnius prolixus* during feeding. (See also Figure 2 – figure supplemental 1). From top to bottom, *T_a_* = 16°C, 21°C, 26°C or 31°C.

| **Source of Variation** | **DF** | **Sum of Squares** | **Mean Square** | **F-value** | **P-value** |
| --- | --- | --- | --- | --- | --- |
| **1- Body part** | 3 | 405.594 | 135.198 | 302.925 | <0.001 |
| **2- Tblood** | 1 | 80.997 | 80.997 | 181.482 | <0.001 |
| **1 x 2** | 3 | 22.165 | 7.388 | 16.554 | <0.001 |
| **Residual** | 16 | 7.141 | 0.446 |  |  |
| **Total** | 23 | 515.897 | 22.430 |  |  |

| **Source of Variation** | **DF** | **Sum of Squares** | **Mean Square** | **F-value** | **P-value** |
| --- | --- | --- | --- | --- | --- |
| **1- Body part** | 3 | 249.821 | 83.274 | 4236.652 | <0.001 |
| **2- Tblood** | 2 | 285.027 | 142.514 | 7250.551 | <0.001 |
| **1 x 2** | 6 | 18.431 | 3.072 | 156.287 | <0.001 |
| **Residual** | 24 | 0.472 | 0.020 |  |  |
| **Total** | 35 | 553.752 | 15.821 |  |  |

| **Source of Variation** | **DF** | **Sum of Squares** | **Mean Square** | **F-value** | **P-value** |
| --- | --- | --- | --- | --- | --- |
| **1- Body part** | 3 | 223.141 | 74.380 | 311.236 | <0.001 |
| **2- Tblood** | 1 | 56.611 | 56.611 | 236.882 | <0.001 |
| **1 x 2** | 3 | 5.118 | 1.706 | 7.138 | 0.003 |
| **Residual** | 16 | 3.824 | 0.239 |  |  |
| **Total** | 23 | 288.693 | 12.552 |  |  |

| **Source of Variation** | **DF** | **Sum of Squares** | **Mean Square** | **F-value** | **P-value** |
| --- | --- | --- | --- | --- | --- |
| **1- Body part** | 3 | 70.527 | 23.509 | 497.810 | <0.001 |
| **2- Tblood** | 1 | 102.589 | 102.589 | 2172.353 | <0.001 |
| **1 x 2** | 3 | 1.931 | 0.644 | 13.630 | <0.001 |
| **Residual** | 16 | 0.756 | 0.047 |  |  |
| **Total** | 23 | 175.803 | 7.644 |  |  |
