# Supplementary material for: Countercurrent heat exchange and thermoregulation during blood-feeding in kissing bugs
Source: eLife. 2017 Nov 21;6:e26107. doi: 10.7554/eLife.26107 (PMC5697934; doi:10.7554/eLife.26107)
Supplement: Transparent reporting form [file elife-26107-transrepform.docx]

***eLife’s* transparent reporting form**

We encourage authors to provide detailed information *within their submission* to facilitate the interpretation and replication of experiments. If you have any questions, please contact us: [editorial@elifesciences.org](mailto:editorial@elifesciences.org).

**Sample-size estimation**

- You should state whether an appropriate sample size was computed when the study was being designed
- You should state the statistical method of sample size computation and any required assumptions
- If no explicit power analysis was used, you should describe how you decided what sample (replicate) size (number) to use

Please outline where this information can be found within the submission (e.g., page numbers or figure legends), or explain why this information doesn’t apply to your submission:

Morphological analysis: Histology was performed on 10 animals (lines 419-420), 14 were scanned in the µCT (line 467) and 16 were filmed in the X-ray Synchrotron (some more than once) (lines 454-455). The 3D reconstruction of the head was based on the best preparations obtained, serving the others for confirming specific features.

Thermographic measures presented in Fig. 2 were performed on a total of 27 individuals, 3 per combination of environment-blood temperatures (lines 375-376).

The expression of HSP70 and HSP90 by qPCR assays was performed on five biological replications per experimental group (line 513, Figure 2E).

**Replicates**

- You should report how often each experiment was performed
- You should include a definition of biological versus technical replication
- The data obtained should be provided and sufficient information should be provided to indicate the number of independent biological and/or technical replicates
- If you encountered any outliers, you should describe how these were handled
- Criteria for exclusion/inclusion of data should be clearly stated
- High-throughput sequence data should be uploaded before submission, with a private link for reviewers provided (these are available from both GEO and ArrayExpress)

Please outline where this information can be found within the submission (e.g., page numbers or figure legends), or explain why this information doesn’t apply to your submission:

Each data, morphological or thermographic, was obtained from a biological replication (i.e. individual). The process of analysis implicated either feeding (thermography, X-ray synchrotron imaging), killing (histology) or exposing the insect to potentially severe conditions (µCT), making impossible a reutilisation of an individual, with the exception of some individuals that could be filmed more than once in the X-ray synchrotron. So, each bug was usually used only once.

Morphological analysis (histology, µCT, X-ray Synchrotron imaging) and reconstructions are based on the best preparations obtained (i.e., good fixation and staining, good contrast, low noise, good resolution, no evident artifacts), serving the other for confirming specific features.

The expression of HSP70 and HSP90 by qPCR assays was performed on five biological replications per experimental group.

**Statistical reporting**

- Statistical analysis methods should be described and justified
- Raw data should be presented in figures whenever informative to do so (typically when N per group is less than 10)
- For each experiment, you should identify the statistical tests used, exact values of N, definitions of center, methods of multiple test correction, and dispersion and precision measures (e.g., mean, median, SD, SEM, confidence intervals; and, for the major substantive results, a measure of effect size (e.g., Pearson's r, Cohen's d)
- Report exact p-values wherever possible alongside the summary statistics and 95% confidence intervals. These should be reported for all key questions and not only when the p-value is less than 0.05.

Details on statistics can be found in lines 398-408 and 512-515, and the output of ANOVA in the Supplementary tables 1. Parametric statistics were applied on data that have previously passed normality and homoscedasticity tests.

Please outline where this information can be found within the submission (e.g., page numbers or figure legends), or explain why this information doesn’t apply to your submission:

(For large datasets, or papers with a very large number of statistical tests, you may upload a single table file with tests, Ns, etc., with reference to page numbers in the manuscript.)

**Additional data files (“source data”)**

- We encourage you to upload relevant additional data files, such as numerical data that are represented as a graph in a figure, or as a summary table
- Where provided, these should be in the most useful format, and they can be uploaded as “Source data” files linked to a main figure or table
- Include model definition files including the full list of parameters used
- Include code used for data analysis (e.g., R, MatLab)
- Avoid stating that data files are “available upon request”

Please indicate the figures or tables for which source data files have been provided:

Raw data are provided as supplementary material: Figure 2- source data 1; Figure 2 – source data 2 and Supplementary file 1).
